# The therapeutic potential of probucol and probucol analogues in neurodegenerative diseases

**DOI:** 10.1186/s40035-024-00398-w

**Published:** 2024-01-22

**Authors:** Arazu Sharif, John Mamo, Virginie Lam, Hani Al-Salami, Armin Mooranian, Gerald F. Watts, Roger Clarnette, Giuseppe Luna, Ryu Takechi

**Affiliations:** 1 Curtin Health Innovation Research Institute, Faculty of Health Sciences, Curtin University, Perth, WA Australia; 2 Perron Institute for Neurological and Translational Research, Perth, WA Australia; 3 School of Public Health, Faculty of Health Sciences, Curtin University, Perth, WA Australia; 4 School of Medicine, Faculty of Health and Medical Sciences, University of Western Australia, Perth, WA Australia; 5 Curtin Medical School, Faculty of Health Sciences, Curtin University, Perth, WA Australia

**Keywords:** Neurodegenerative disease, Probucol, Probucol analogues, Inflammation, Blood–brain barrier, Oxidative stress, Inflammation, Amyloid-beta

## Abstract

Neurodegenerative disorders present complex pathologies characterized by various interconnected factors, including the aggregation of misfolded proteins, oxidative stress, neuroinflammation and compromised blood–brain barrier (BBB) integrity. Addressing such multifaceted pathways necessitates the development of multi-target therapeutic strategies. Emerging research indicates that probucol, a historic lipid-lowering medication, offers substantial potential in the realm of neurodegenerative disease prevention and treatment. Preclinical investigations have unveiled multifaceted cellular effects of probucol, showcasing its remarkable antioxidative and anti-inflammatory properties, its ability to fortify the BBB and its direct influence on neural preservation and adaptability. These diverse effects collectively translate into enhancements in both motor and cognitive functions. This review provides a comprehensive overview of recent findings highlighting the efficacy of probucol and probucol-related compounds in the context of various neurodegenerative conditions, including Alzheimer’s disease, Parkinson’s disease, Huntington’s disease, and cognitive impairment associated with diabetes.

## Introduction

Neurodegenerative diseases (NDs), including Alzheimer’s disease (AD), Parkinson's disease (PD) and Huntington’s disease (HD), are marked by the loss of specific neuronal cell function. In addition, neurodegeneration can result from primary metabolic or vascular disorders such as diabetes and stroke [1, 2]. The global health burden of NDs is escalating due to the aging populations. Evidently, the development of effective pharmacotherapies for the treatment of NDs increasingly stands as a contemporary global health imperative in the field of neuromedicine.

NDs typically manifest in response to central nervous system (CNS) insults, chronic or transient disruptions of the cerebral microvasculature, the accumulation of disordered proteins in the form of protein or lipid inclusion bodies or lipofuscin aggregates, inflammation, and oxidative stress [3]. Inflammation, characterized by astrogliosis, may foster the parenchymal extravasation of circulating leukocytes, triggering a cyclical inflammatory process. Subsequently, the exaggerated production or inadequate sequestration of oxygen-derived free radicals and non-radical species released via the respiratory burst of inflammatory glial cells, jeopardizes cell viability through intricate pathways, which may encompass mitochondrial fusion [4], the disruption of intracellular ion homeostasis [5], or cell autophagy/mitophagy [6]**.**

Activation of the peripheral innate immune system can also exert central effects that might be pertinent to the onset and progression of NDs. This is because cytokines, chemokines and damage-associated soluble mediators of systemic inflammation can penetrate brain parenchyma through the bloodstream. For example, in systemic lupus erythematosus, a chronic relapse-remitting autoimmune disease affecting multiple organs beyond the brain, antinuclear antibodies are also implicated centrally and associated with neurodegeneration [7]. In metabolic disorders such as poorly controlled diabetes, the risk of NDs is elevated through a heightened systemic cytokine axis and interactive effects with heightened innate immunity. Furthermore, Godbout et al. demonstrated that the activation of innate immunity worsens significantly with aging [8].

In a seminal review by Cobley et al. [9], the authors described how the brain might be particularly sensitive to vascular inflammation and oxidative stress due to factors including the enrichment of oxidizable polyunsaturated fatty acids, accumulation of calcium and glutamate, substantial mitochondrial abundance, significant aerobic metabolism coupled with modest antioxidant defence, a notable presence of redox-active transition metals (copper and iron) and a susceptibility to neurotransmitter auto-oxidation and RNA oxidation. This significant body of evidence, from cellular and preclinical studies to clinical and population research highlighting the central role of neurovascular inflammation and oxidative stress in the evolution of NDs, is propelling new lines of research and drug development. Contemporary approaches being explored encompass a wide array of mitoprotective agents, modulation of inflammatory transcription factors, small-molecule modulators of mitochondrial function, neurotrophic factor therapies, bioenergetic modulation, gene therapy, RNA-modulating treatments such as antisense oligonucleotides, and multi-target polypharmacological strategies [3].

Intriguingly, few studies have considered the potential repurposing of historically used drugs for new applications in NDs, possibly reflecting challenges related to commercialization. The latter suggests an extraordinary opportunity to discover new treatments for NDs that have a well-established safety and tolerability profile. In this review, we examine the accumulating body of evidence suggesting that the historical cholesterol-lowering drug, probucol, possesses multiple polypharmacological cellular effects that could be highly relevant to the progression of NDs. This review will focus on the potential beneficial effects of probucol in the treatment of AD, PD, HD, and diabetes-associated cognitive impairment.

## Historic probucol studies in cardiovascular disease

Probucol is a bisphenol compound that has been used historically to lower cholesterol and treat cardiovascular disease and xanthomas, as reviewed extensively by Yamashita et al. [10]. Its multifaceted properties including its abilities to reduce monocyte adherence and lower lipid levels, coupled with potent antioxidant and anti-inflammatory activities, contribute to its profound therapeutic outcomes.

Nevertheless, in the context of the discussed benefits of probucol in cardiovascular risk reduction, it is important to acknowledge potential concerns associated with its use. Specifically, it has been reported that probucol has the potential to prolong the QT interval, raising considerations regarding its impact on cardiac electrophysiology [11]. Moreover, another aspect to consider is the observed reduction in high-density lipoprotein (HDL) cholesterol associated with probucol use. While HDL cholesterol has traditionally been considered as having a protective role against cardiovascular disease, the reduction in HDL cholesterol by probucol may, in fact, reflect an accelerated rate of reverse cholesterol transport. Probucol, via activation of cholesteryl ester transfer protein, increases the transfer of cholesterol esterified from HDL to apoB-containing lipoproteins [12, 13]. Moreover, it enhances the expression of hepatic scavenger receptor class B type I which promotes the excretion of cholesterol into the bile and feces [14, 15]. The subsequent reduction of HDL size and cholesterol content, increases its capacity for cholesterol efflux and thereby accelerates reverse cholesterol transport.

Despite these potential concerns, probucol continues to be utilized as an adjunct therapy in Southeast Asian nations, including Japan, to address residual cardiovascular risk [16].

## Potential properties of probucol for neurodegenerative disease risk reduction

### Effects of probucol on cerebral capillary dysfunction

The high rate of cerebral metabolism necessitates the presence of an extensive vascular capillary network. To safeguard the brain against exposure to potentially toxic components of the blood, it is equipped with a highly specialized vascular structure known as the blood–brain barrier (BBB) [17]. The BBB serves as both a physical and a metabolic barrier between the brain and the systemic circulation, selectively permitting the passage of molecules essential for maintaining the healthy metabolic activity of the brain. Several types of cells contribute to the BBB function, including endothelial cells (ECs), pericytes, and astrocytes [18]. ECs form a tightly interconnected first line of defence through the presence of tight junction proteins. Pericytes, embedded within the vascular basement membrane, cover the abluminal side of ECs, providing tone and vessel stability. Positioned between the pericytes and neurons, astrocytes are key regulators of BBB's permeability and play a pivotal role in BBB repair and maintenance.

Over the past few decades, an extensive body of literature has convincingly demonstrated that changes in BBB integrity and function represent a common early feature in the development of many NDs. These changes may encompass reduced expression of tight junction proteins [19–21], increased proliferation of neurotoxic reactive astrocytes [22], loss of pericytes [23–25], degeneration of astrocytes and ECs [26, 27] and abnormal expression of transporter proteins such as GLUT1 isoform of glucose transporter [28, 29], low-density lipoprotein receptor-related protein (LRP)-1 [30], receptor of advanced glycation end products [31], and P-glycoprotein [23, 32]. The evolution of cerebral capillary dysfunction is increasingly recognized as preceding neurodegeneration in several NDs, suggesting a shared contributory factor for onset and progression. Consequently, pharmacological strategies aimed at preserving BBB integrity may hold significant potential to delay the onset and progression of NDs.

Probucol has been demonstrated to have protective effects on the BBB. In an ischemia-induced mouse model of BBB dysfunction, Nakagawa et al. reported that probucol, through the attenuation of sphingosine 1-phosphate signalling and inactivation of signal transducer and activator of transcription 3, preserved the proper localization of tight junction protein in ECs, thereby reducing the leakage of small molecules into the brain parenchyma [33]. Additionally, probucol may protect the BBB by enhancing EC function. In a study by Takase et al., a combination therapy with probucol and cilostazol resulted in improved endothelial function in patients with silent lacunar cerebral infarcts [34]. Moreover, in an in vitro model of cerebral endothelial dysfunction, probucol exhibited an inhibitory effect on the mRNA expression of proapoptotic genes such as caspase-3 and Bax while promoting the expression of B-cell lymphoma 2 (Bcl-2) and endothelial nitric oxide synthase, genes involved in cell survival [35]. The protective effects of probucol on the BBB may also be in part attributed to the reduction of cholesterol overload within ECs. Elevated cholesterol levels promote excessive mitochondrial and endoplasmic reticulum stress in ECs, which leads to oxidative stress and inflammation. The oxidative stress further compromises the structure and localization of tight junction proteins, including zonula occludens (ZO) proteins ZO-1 and ZO2 as well as occludin [36, 37], and upregulates the activity of matrix metalloproteinases, which degrade tight junction proteins [38]. Furthermore, the activation of the nuclear factor-kappa B (NF-kB) pathway by reactive oxygen species (ROS) can increase BBB permeability by inducing cytoskeletal rearrangement in ECs [39]. Chronically elevated oxidative stress can further trigger a proinflammatory response in ECs, exacerbating BBB dysfunction. Elevated levels of inflammatory mediators such as interleukin (IL)-1β, IL-6, IL-9, IL-17, CLL2, tumor necrosis factor (TNF)-α, and interferon (IFN)-γ have been shown to induce BBB dysfunction by downregulating the expression of tight junction proteins and disrupting their translocation [40–45]. The BBB-protective properties of probucol appear to involve an anti-inflammatory axis. Indeed, in lipotoxicity-induced mouse models of BBB dysfunction and in aged mice maintained on saturated fatty acids, probucol has demonstrated its ability to safeguard the BBB [46, 47].

### Effects of probucol on neurovascular inflammation

Chronic activation of microglia and the infiltration of systemically derived leukocytes into the brain parenchyma are strongly implicated as triggering and/or amplifying factors in NDs, including AD [48], PD [49], and HD [50].

The potential therapeutic effects of probucol in modulating inflammation in NDs have not been extensively explored. Nevertheless, studies conducted in rodent models of aging have yielded promising results, demonstrating that probucol significantly attenuates markers of neuroinflammation and neurodegeneration [46, 51].

Champagne et al. reported that probucol significantly reduced glial activation in aged rats, coinciding with an increase in cerebrospinal fluid (CSF) apolipoprotein E (apoE) level [52]. Notably, low levels of apoE in CSF are commonly observed in patients with AD [53]. In murine models of BBB dysfunction and ischemic brain injury, probucol was shown to inhibit the expression of oxidation-sensitive inflammatory mediators such IL-1 [51], and vascular cell adhesion molecule-1 [54]. Moreover, probucol effectively suppressed the mRNA expression of pro-inflammatory mediators including IL-1β, IL-6, nitric oxide, and prostaglandin E2 in BV2 and primary microglial cells and reduced expression of nitric oxide synthase and cyclooxygenase-2 in ischemic mouse brains through inhibition of activator protein-1, NF-kB and the mitogen-activated protein kinase pathway [55]. Oxidative stress is a consequence of the inflammatory cascade, but the damage to lipids, proteins, or cells induced by oxidative stress can in turn perpetuate the cycle of inflammation. Probucol may indirectly mitigate inflammation by reducing oxidative damage.

The collective evidence to date suggests that probucol holds promise as a protective agent against NDs by attenuating neuroinflammation through mechanisms involving increased apoE levels and potentially the inhibition of oxidation-mediated pathways of inflammation.

### Effects of probucol on oxidative stress

Reactive nitrogen and oxygen species are natural by-products of metabolic activity. When they are present in controlled quantities, they serve crucial roles as regulators of cellular growth and function, acting as pivotal mediators of cell signalling, immune responses, and the relaxation of smooth muscles [56]. However, an excess of these reactive molecules leads to oxidative stress, resulting in harmful damage to cellular function and eventual cell death.

Probucol has the potential to mitigate the oxidative cascade both directly and indirectly. It acts directly by serving as a single electron donor and quenching singlet oxygen [57]. Additionally, probucol indirectly stimulates the activity and increases the protein level of key antioxidant enzymes. In a mouse model of neurodegeneration induced by D-galactose, probucol administration effectively eliminated the accumulation of ROS and malondialdehyde, by enhancing the mRNA expression and protein level of antioxidant enzymes like superoxide dismutase, glutathione peroxidase and heme oxygenase-1 [58]. This positive effect of probucol was attributed to its influence on the nuclear factor erythroid 2-related factor 2 (Nrf2)/kelch-like ECH-associated protein 1 (Keap1) pathway, a major regulator of the antioxidant response. Probucol facilitated the expression of Nrf2 and promoted its translocation into the nucleus by facilitating the dissociation of the Nrf2/Keap1 complex. Similarly, Zhou et al. reported that probucol activates the Nrf2/antioxidant response element pathway and mammalian target of rapamycin signalling pathway, leading to the inhibition of inflammation and neuronal cell apoptosis following spinal cord injury in rats [59, 60].

Moreover, studies have shown that probucol enhances the activity of antioxidant enzymes. For instance, research conducted by Santos and colleagues demonstrated that probucol positively regulates the activity of glutathione peroxidase-1, an enzyme crucial for detoxifying peroxides in the CNS, without affecting its expression level, thereby protecting neuronal cells against hydroperoxides [61].

In summary, probucol may mitigate neuronal cell damage and apoptosis and thereby prevent the onset of NDs by effectively enhancing the production of antioxidants and inhibiting oxidative stress pathways.

### Effects of probucol on neural survival and neuroplasticity

Probucol holds significant promise in mitigating neurodegeneration through its modulation of redox, inflammatory and cerebral microvascular pathways. Furthermore, it directly functions as a neural survival and apoptosis regulator. In a D-galactose-induced mouse model of aging, probucol effectively inhibited the hippocampal protein levels of p53 and p16, two key regulators of cell senescence and apoptosis [62]. This research also unveiled 70 genes regulated by probucol, including those involved in neuronal survival (*Pkib*), protection against oxidative stress (*Gstp2*), regulation of mitochondrial respiration, and maintenance of lipid and glucose metabolism (*Pask*). The authors also reported probucol-mediated improvement of long-term potentiation, increased spine density and dendritic branches, and elevated expression of the postsynaptic scaffolding protein 95 in the CA1 region of the hippocampus, all of which contribute to improved memory and learning.

Similarly, in a rat model of spinal cord injury, probucol successfully mitigated neural cell apoptosis by upregulating the expression of the anti-apoptotic protein Bcl-2 while downregulating the pro-apoptotic proteins such as Caspase-3, Caspase-9, and Bax [59]. Furthermore, the study reported the probucol-induced inactivation of mammalian target of rapamycin signalling pathway and enhanced levels of autophagy-related proteins such as Beclin-1 and LC3B, which are essential for neurodevelopment and neural survival.

**Fig. 1:**
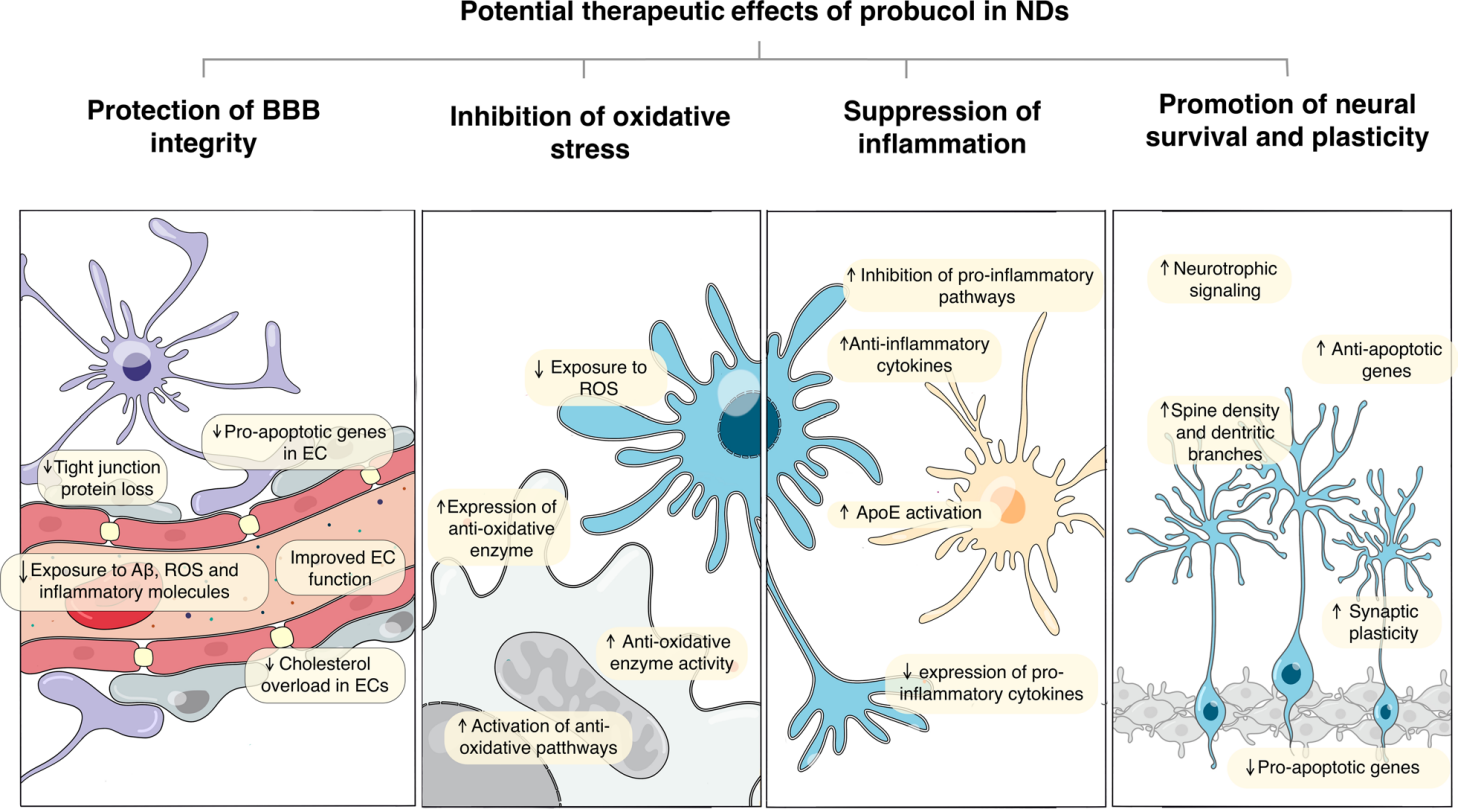
Schematic summary of the effects of probucol in protecting BBB (**a**), reducing oxidative stress (**b**), alleviating inflammation (**c**), and promoting neuronal survival and plasticity (**d**)

Collectively, these findings suggest that probucol may offer therapeutic benefits for NDs by effectively enhancing neural survival and plasticity. The ability of probucol to modulate redox, inflammatory and cerebral microvascular pathways, coupled with its direct impact on neural survival and synaptic plasticity, makes it a promising candidate for the treatment of NDs (Fig. 1.)

## Emerging evidence of efficacy of probucol in AD

The aggregation of misfolded proteins stands as a hallmark feature of various NDs. The formation of senile plaques rich in amyloid beta (Aβ) and neutral lipids is strongly implicated in AD pathogenesis. While the precise origin of these Aβ and lipid aggregates remains unclear, mounting evidence suggests that Aβ, secreted into circulation by peripheral lipogenic organs as a regulator of nascent triglyceride-rich lipoproteins, may play a pivotal role in this process [63–66]. This hypothesis gains compelling support from studies involving genetically engineered mice exclusively synthesizing human Aβ in the liver, where premature age-associated BBB disruption, increased parenchymal neutral lipid inclusion bodies, astrogliosis, neurovascular inflammation and oxidative stress were observed. Additionally, some studies suggest that certain dietary lipids particularly atherogenic saturated fatty acids, and the apoE4 genotype (an independent risk factor for AD), may have interactive effects that modulate the lipoprotein-Aβ homeostasis and by extension, AD risk [67–70].

Multiple studies propose that probucol may mitigate Alzheimer's risk through the modulation of the peripheral metabolism of lipoprotein-Aβ. In wild-type mouse models, probucol preserves capillary integrity, prevents extravasation of lipoprotein-Aβ into brain parenchyme, and suppresses astrogliosis and neurovascular inflammation, all profoundly inhibiting the secretion of lipoprotein amyloid, a finding consistent with a causal mechanism [47, 71, 72]. Probucol has also been shown to accelerate the clearance of lipoproteins ordinarily enriched in Aβ from the bloodstream, further reducing the cerebrovascular exposure [73]. This may be achieved via an increased abundance of apoE, the primary binding ligand for receptor-mediated clearance of post-hydrolyzed triglyceride-rich lipoproteins which are the primary chaperone lipoprotein of Aβ [52]. Moreover, Santos et al. reported that administering probucol (10 mg/kg, intraperitoneally) to mice for 2 weeks reduced lipid peroxidation, increased acetylcholinesterase activity and improved synaptic deficits and cognitive impairment caused by a single intraventricular injection of Aβ_1–40_ [74]. Probucol may positively modulate brain Aβ homeostasis by stimulating the expression of LRP on capillary endothelium. This facilitates the efflux of Aβ from the brain to the bloodstream, potentially reducing Aβ burden [75–77]. Furthermore, in vitro studies have indicated that the apoE/Aβ complex may delay fibril formation, thus preventing toxic interactions with neuronal plasma membranes [78, 79]. The stimulatory effect of probucol on the expression of apoE may diminish the propensity for Aβ fibril formation.

The positive effects of probucol on the expression of synaptic markers, including synaptophysin and synaptosomal-associated protein 25, have been reported in a mouse model of AD and in a rat model of aging, respectively [52, 74]. These markers are crucial for neural processes such as neurotransmission, synaptic plasticity and integrity and they play important roles in learning and memory. In a toxin-induced mouse model of cognitive impairment, probucol prevented cognitive decline, suppressed the increase in acetylcholinesterase activity, and reduced oxidative stress by promoting the activity of glutathione peroxidase (GPx) and glutathione reductase (GR) [80].

A pilot study involving 12 participants has suggested the potential memory-enhancing effects of probucol in AD [81]. The study showed that 6-month probucol treatment (500 mg b.i.d.) led to stabilisation of cognitive function in individuals with mild to moderate AD. Another study published in 2022 outlines a contemporary double-blind, placebo-controlled, randomized phase II trial investigating the efficacy of probucol on cognitive function in AD [82]. The study aims to investigate if probucol has the potential to attenuate cognitive decline, delay brain atrophy, and reduce cerebral amyloid burden in AD.

## Potential efficacy of probucol in PD

PD is characterized by the loss of dopaminergic neurons and the formation of protein inclusions enriched in alpha-synuclein protein. In a study published in 2013, researchers investigated the effects of probucol on the vulnerability of striatal dopaminergic neurons to oxidative stress in an in vivo PD model. The study revealed that probucol provides significant protection against 6-hydroxydopamine-induced hyperlocomotion, mitigates the decrease in tyrosine hydroxylase levels and prevents striatal lipid peroxidation and oxidative stress by reducing the catalase activity while concurrently increasing GPx and GR activity [83].

Emerging evidence suggests that disruptions in mitophagy are causally associated with the loss of dopaminergic neurons [84]. Mitophagy is a process in which damaged mitochondria are cleared, preventing neuronal cytotoxicity and preserving the survival of dopaminergic neurons. By employing an artificial intelligence platform to study candidate mitophagy enhancer molecules, probucol was validated across several mitophagy assays. In Zebrafish and *Drosophila* models of mitochondrial damage, probucol improves the survival of dopaminergic neurons and locomotor function. The function of probucol depends on the expression of the ATP-binding cassette transporter A1 (ABCA1), which negatively regulates mitophagy following mitochondrial damage. ABCA1 is primarily considered to function in maintaining cellular cholesterol and phospholipid homeostasis, serving as a cellular efflux portal enzyme. In vivo studies found that autophagosome and lysosomal markers are elevated with probucol treatment, while lipid droplet formation is suppressed [85].

Environmental toxins such as paraquat and rotenone, as well as bacterial metabolites produced by *Streptomyces venezuelae*, can induce and accelerate age- and dose-dependent dopaminergic neurodegeneration via mitochondrial disruption. Using an ex vivo assay of *C. elegans* extracts, researchers found that the bacterial metabolite caused cell death through exaggerated production of ROS and exhibited additive effects with environmental toxins. In this model, probucol was found to fully rescue the metabolite/toxin-induced dopaminergic neurodegeneration [86].

## Potential efficacy of probucol in HD

HD is an autosomal dominant inherited neurodegenerative disorder caused by a trinucleotide expansion in the HTT gene, resulting in an extended polyglutamine tract in the protein huntingtin. In HD, striatal and cortical neurons prematurely undergo cell death and the mechanisms underlying this cell death are believed to involve oxidative stress, excitotoxicity and disrupted energy metabolism. While HD is commonly categorized as a movement disorder, it is essential to note the significant cognitive and neuropsychiatric symptoms that also manifest clinically [87].

A frequently used model for studying HD involves the irreversible inhibition of the mitochondrial enzyme succinate dehydrogenase using 3-nitropropionic acid (3-NP). In rat striatal slices exposed to 3-NP and quinolinic acid as an excitotoxic model of HD, probucol effectively prevented the formation of ROS and lipid peroxidation [88].

In vivo studies utilizing the 3-NP model of HD in rats showed that probucol treatment for 2 months protected against behavioural and striatal biochemical changes induced by 3-NP administration. Specifically, probucol decreased striatal oxidative stress while simultaneously increasing the activity of GPx [89].

Positive behavioural effects were also demonstrated in other studies using the YAC128 transgenic mouse model of HD. During the early- to mild-symptomatic stages of disease progression, treatment with probucol up to 5 months of age in YAC128 mice reduced the occurrence of depressive-like behaviour in early- and mild-symptomatic YAC128 mice. These findings collectively support the notion that probucol may hold relevance when extrapolated to human neurodegenerative processes involving mitochondrial dysfunction and suggest that GPx may mediate the beneficial effects of probucol [90].

## Potential efficacy of probucol in supporting cognitive function in diabetes

Type 2 diabetes (T2D) stands as the most prevalent metabolic disease, affecting over half a billion individuals worldwide [91]. Numerous cross-sectional and longitudinal studies have unequivocally shown that the prevalence of dementia among patients with T2D significantly surpasses that of age-matched non-diabetics [92–94]. Neuroimaging studies have further revealed cerebrovascular disruptions, gray and white matter atrophy, loss of brain connectivity, and metabolic alterations that bear resemblance to early-stage AD but preceding the development of frank amyloidosis [95–97].

In T2D, hyperglycemia and insulin resistance in diabetes are believed to initiate and exacerbate the generation of ROS, impair the antioxidant system, and disrupt mitochondrial function—a milieu reminiscent of other NDs, albeit initiated through distinct pathways [98–101].

In diabetes models, probucol has been observed to positively modulate glucose homeostasis, enhance pancreatic beta cell function, and improve insulin sensitivity [102, 103]. Moreover, in rodent models of diabetes, probucol mitigates complications such as nephropathy [104], peripheral neuropathy [105] and retinopathy [106, 107] through amelioration of REDOX homeostasis.

In pre-diabetic mice, probucol has demonstrated the ability to reduce plasma levels of pro-inflammatory cytokines like IL-6, TNF-α, and IFN-λ, while simultaneously enhancing the expression of the anti-inflammatory cytokine IL-10 [108, 109].

Additional studies have shown that probucol effectively suppresses neurovascular inflammation, restores the expression of tight junction proteins like Occludin-1 and ZO-1, and prevents BBB leakage in a streptozotocin-induced model of type 1 diabetes. These actions are associated with significant attenuation of neurodegeneration and cognitive dysfunction [51].

## Putative efficacy of probucol analogues

### Succinobucol

**Fig. 2:**
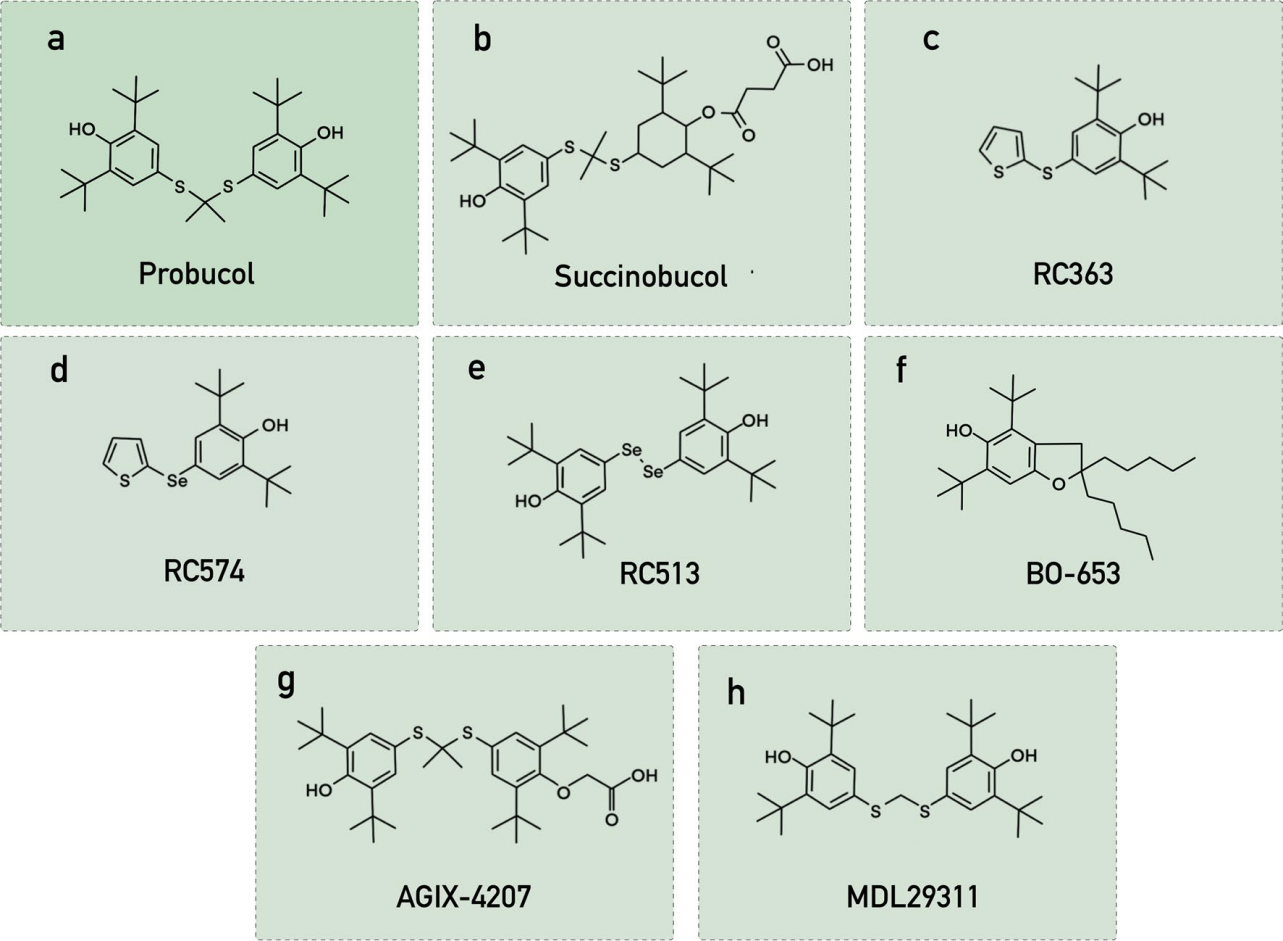
Chemical structures of probucol and probucol analogues

Succinobucol (Fig. 2b), the monosuccinate ester of probucol, faced setbacks in clinical trials targeting acute coronary syndrome, but promising studies in neurodegeneration models have underscored its potential as an antioxidant and anti-inflammatory agent in NDs.

Succinobucol administration in an MPTP-induced behavioural impairment model of PD improved non-motor behavioural parameters and preserved immunoreactivities of tyrosine hyrdorxylase and dopamine transporter in the substantia nigra and striatum while suppressing astroglial activation and increasing IL-6 level [110]. Ribeiro et al. noted partial protection by succinobucol against toxin-induced forelimb inability in a PD mouse model [111]. These findings suggest the potential of succinobucol to counteract early non-motor symptoms, neurodegeneration and neuroinflammation in the nigrostriatal pathway.

In an in vitro experimental model of HD, both probucol and succinobucol protected against the toxin-induced lipid peroxidation and increased ROS generation, but only succinobucol prevented mitochondrial dysfunction [112]. In a cultured neuroblastoma (SH-SY5Y) cell model, succinobucol significantly elevated GSH levels by stimulating the translocation of Nrf2 into the nucleus, and enhanced glutamate cysteine ligase mRNA expression and activity, offering protection against mitochondrial dysfunction-derived oxidative stress [113].

### RC363 and RC574

Bueno et al. developed two novel probucol analogues, RC363 and RC574 (Fig. 2c, d) [114]**.** These compounds demonstrated neuroprotective activity against glutamate-mediated oxidative cell death in an immortalized murine hippocampal cell line (HT22). In comparison to probucol, both analogues exhibited greater efficiency in inhibiting mitochondrial superoxide anion production while increasing the activity of antioxidant enzymes, including GPx-1. Another study showed significant cytoprotective effects of the two compounds against glutamate-, NSO- or RSL3-induced ferroptosis in the HT22 cell line, which were more pronounced than the effect of probucol [115]. Further analysis of RC574, also known as C2, in a rat model of ischemic stroke demonstrated its effectiveness in improving motor-related behavioural deficits and reducing reactive astrocytes in the striatum [116].

### RC513

RC513 (Fig. 2e), an organoselenium derivative of probucol, has displayed substantial promise in inhibiting oxidative toxicity. RC513 protected cultured HT22 cells against t-BuOOH-induced mitochondrial damage by modulating the expression and activity of peroxide detoxification proteins, such as GPx-1 and GPx. Similar protective effects of RC513 have been observed against methylmercury-induced neurotoxicity [117].

### BO-653

BO-653 (Fig. 2f), structurally akin to probucol and α-tocopherol, exhibits tenfold greater antioxidant potency than probucol [118, 119]. It demonstrated superior antiatherosclerotic effects in animal models without affecting high-density lipoprotein levels [118–120]. However, following its failure in a phase II clinical trial for atherosclerosis treatment and prevention of post-angioplasty restenosis, further investigations were halted [121].

### AGIX-4207

AGIX-4207 (Fig. 2g), a more bioavailable analogue of probucol, displays increased efficacy in reducing intracellular ROS levels despite lacking one of probucol's redox centres. The compound effectively inhibited the LPS-induced secretion of pro-inflammatory cytokines and reduced the levels of endothelial cell adhesion molecules [122, 123]. Similarly, DTBP, a vaso-protective probucol analogue, exhibits anti-inflammatory and antioxidative effects similar to probucol [124]

### MDL29311

The antioxidant MDL29311 (Fig. 2h), a probucol analogue with metabolic effects akin to the antidiabetic drug metformin, directly reduces plasma glucose levels without stimulating insulin secretion [125]. It also lowers plasma triglycerides potentially through promoting lipid hydrolysis by capillary endothelial lipases [126].

## Bioavailability of probucol

Several nanotechnology-based drug delivery strategies have been employed to enhance the absorption and effectiveness of probucol. Notably, nanoencapsulation of probucol using sodium alginate leads to elevated levels of probucol in plasma and facilitates its absorption in the brains of wild-type mice maintained on high-fat diet. This, in turn, leads to a significant reduction of neuroinflammation and neuronal cell death [109]. Furthermore, researchers have explored co-encapsulation approaches, pairing probucol with bile acids as permeation-enhancing agents. For instance, sodium-alginate microencapsulation of probucol alongside lithocholic acid, a secondary bile acid, demonstrated improved drug absorption, increased bioavailability, and enhanced therapeutic outcomes [127]. Similarly, the combination of probucol with and without ursodeoxycholic acid, through alginate-Eudragit microencapsulation, resulted in increased pharmacological activity. Importantly, this method, while not altering systemic probucol levels, reduced its absorption by cardiac tissue in prediabetic mice [108]. This reduction in cardiac muscle absorption may contribute to minimizing the potential cardiotoxic effects associated with probucol treatment.

## Challenges for the use of probucol or derivatives in NDs

While the promising findings discussed in this review underscore the potential therapeutic benefits of probucol and its derivatives in addressing NDs, several challenges must be acknowledged and addressed before their wide clinical application. First, the translation of preclinical success to human trials necessitates a thorough understanding of the pharmacokinetics, optimal dosage, and potential side effects of probucol in diverse populations. Variability in individual responses, potential drug interactions and long-term safety profiles represent critical considerations that merit comprehensive investigation.

Furthermore, the intricate nature of NDs demands a nuanced approach in therapeutic development. The efficacy of probucol in targeting multiple pathways is encouraging, yet the specific mechanisms underlying its neuroprotective effects require further elucidation. Unravelling the molecular intricacies will aid in refining treatment strategies and tailoring interventions to specific subtypes of neurodegenerative conditions.

The BBB poses another challenge, as enhancing its integrity is a double-edged sword. Striking the right balance between the barrier against harmful agents and allowing the passage of therapeutic compounds remains a delicate task. Moreover, the chronic and progressive nature of NDs necessitates a sustained, long-term treatment approach, raising concerns about patient compliance and potential adverse effects over extended periods.

As the field moves forward, collaborative efforts between researchers, clinicians, and pharmaceutical entities will be pivotal in overcoming these challenges. Rigorous clinical trials with well-defined endpoints, robust methodologies and diverse participant cohorts are imperative to validate the translational potential of probucol in human NDs. While the road ahead is challenging, the compelling evidence presented in this review underscores the importance of continued exploration and refinement of probucol-based therapeutic approaches for the benefit of individuals afflicted by these devastating disorders.

## Conclusion

As the global health burden of NDs continues to rise, the need for effective pharmacotherapies becomes increasingly urgent. The concept of repurposing compounds such as probucol with well-established safety and tolerability profiles for novel applications offers a promising opportunity for expediting drug development processes. The body of evidence presented here highlights the multifaceted therapeutic potential of probucol that ranges from preserving blood–brain barrier integrity and attenuating neurovascular inflammation to mitigating oxidative stress and enhancing neural survival and neuroplasticity. While such promising preclinical and in vitro data provide a strong foundation for its potential efficacy, clinical trials such as the ongoing randomized phase II study that is examining probucol’s effects on cognitive function in AD, are warranted to further validate its clinical use.

## Data Availability

Not applicable.

## Abbreviations

NDs: Neurodegenerative disease
AD: Alzheimer’s disease
PD: Parkinson’s disease
HD: Huntington's disease
CNS: Central nervous system
HDL: High-density lipoprotein
BBB: Blood–brain barrier
ECs: Endothelial cells
LRP: Low-density lipoprotein receptor-related protein-1
Bcl-2: B-cell lymphoma 2
ZO: Zonula occludens
ROS: Reactive oxygen species
IL: Interleukin
TNF: Tumor necrosis factor
IFN: Interferon
CSF: Cerebrospinal fluid
ApoE: Apolipoprotein E
Nrf2: Nuclear factor erythroid 2-related factor 2
Keap1: Kelch-like ECH-associated protein 1
Aβ: Amyloid beta
GPx: Glutathione peroxidase
GR: Glutathione reductase
ABCA1: ATP-binding cassette transporter A1
3-NP: 3-Nitropropionic acid
T2D: Type 2 diabetes
